# Doxorubicin-Induced Platelet Activation and Clearance Relieved by Salvianolic Acid Compound: Novel Mechanism and Potential Therapy for Chemotherapy-Associated Thrombosis and Thrombocytopenia

**DOI:** 10.3390/ph15121444

**Published:** 2022-11-22

**Authors:** Wenjing Ma, Zackary Rousseau, Sladjana Slavkovic, Chuanbin Shen, George M. Yousef, Heyu Ni

**Affiliations:** 1 Department of Laboratory Medicine and Pathobiology, University of Toronto, Toronto, ON M5S 1A1, Canada; 2 Department of Laboratory Medicine, LKSKI-Keenan Research Centre for Biomedical Science, St. Michael’s Hospital, Toronto Platelet Immunobiology Group, Toronto, ON M5B 1W8, Canada; 3 School of Medicine and Pharmacy, Ocean University of China, Qingdao 266003, China; 4 Department of Medicine, University of Toronto, Toronto, ON M5S 1A1, Canada; 5 Canadian Blood Services Centre for Innovation, Toronto, ON M5G 2M1, Canada; 6 Department of Physiology, University of Toronto, Toronto, ON M5S 1A1, Canada

**Keywords:** doxorubicin, salvianolic acid C, platelet activation, platelet clearance, cancer-platelet crosstalk, tyrosine phosphorylation

## Abstract

Doxorubicin (Dox) is a widely utilized chemotherapeutic; however, it carries side effects, including drug-induced immune thrombocytopenia (DITP) and increased risk of venous thromboembolism (VTE). Currently, the mechanisms for Dox-associated DITP and VTE are poorly understood, and an effective inhibitor to relieve these complications remains to be developed. In this study, we found that Dox significantly induced platelet activation and enhanced platelet phagocytosis by macrophages and accelerated platelet clearance. Importantly, we determined that salvianolic acid C (SAC), a water-soluble compound derived from Danshen root traditionally used to treat cardiovascular diseases, inhibited Dox-induced platelet activation more effectively than current standard-of-care anti-platelet drugs aspirin and ticagrelor. Mechanism studies with tyrosine kinase inhibitors indicate contributions of phospholipase C, spleen tyrosine kinase, and protein kinase C signaling pathways in Dox-induced platelet activation. We further demonstrated that Dox enhanced platelet-cancer cell interaction, which was ameliorated by SAC. Taken together, these findings suggest SAC may be a promising therapy to reduce the risk of Dox-induced DITP, VTE, and the repercussions of amplified platelet-cancer interaction in the tumor microenvironment.

## 1. Introduction

Platelets are small, anucleate blood cells constitutively generated in the bone marrow and/or lungs, to enter circulation [1,2]. Physiologically, platelet adhesion at sites of vascular injury, activation, and aggregation is pivotal to forming a hemostatic plug to cease bleeding [1,3]. Platelet activation is achieved following tethering to exposed extracellular matrix ligands such as collagen and VWF, and it also occurs via soluble agonists such as ADP, thrombin, or TxA2 [4]. Upon activation, platelets release intracellular α and dense granules containing a variety of factors, including P-selectin in the former and ADP, calcium, or TxA2 in the latter. During activation, integrin αIIbβ3, the major adhesion receptor of platelets, shifts to an active conformation, with greater ligand binding affinity mediating both Fibrinogen (Fg)-dependent and -independent platelet aggregation [5,6,7,8]. In addition to creating an initial plug at sites of vascular injury (i.e., the first wave of hemostasis), platelets further produce a negatively charged phosphatidylserine (PS)-rich surface to augment cell-based thrombin generation, enhancing blood coagulation (i.e., the second wave of hemostasis) [9,10]. 

Though platelet activation and aggregation are necessary events of normal hemostasis, they can nevertheless swiftly become deleterious such as in pathological conditions where vessels become occluded, including myocardial infarction, stroke, and venous thromboembolism (VTE), or be exploited such as in cancer-associated thrombosis (CAT) [11,12,13,14]. CAT represents the second leading cause of death for patients suffering from malignant tumors, as tumors are capable of inducing coagulation as well as directly activating platelets, accentuating the integral reciprocity between cancer and platelets [15,16,17]. Moreover, another contributing factor to high rates of thrombotic mortality in cancer patients may arise from chemotherapy drugs with off-target effects inducing platelet activation and aggregation, highlighting the gravity of platelet hyperactivity and aggregation in an already fragile system, as approximately 20% of chemotherapy patients develop thrombi following treatment [18,19]. Beyond hemostasis and thrombosis, platelets are appreciated for broader pathophysiological roles with implications in tumorigenesis, angiogenesis, and metastasis [1]. 

Doxorubicin (Dox) is a standard-of-care anthracycline chemotherapeutic agent that has been effectively applied to combat multiple types of cancer. Though a potent antineoplastic agent, Dox is limited due to side effects including cardiotoxicity and nephrotoxicity, drug-induced immune thrombocytopenia (DITP), and increased risk of developing venous thromboembolism (VTE) [20]. The precise effects of Dox on platelet function remain controversial; one study indicated that Dox enhanced low-dose agonist-induced aggregation [21], whereas another reported that Dox inhibited agonist-induced aggregation [22]. A separate study found that Dox had no effect on aggregation but promoted the platelet pro-coagulant state [23], while yet another report suggested that Dox does not induce platelet activation and impairs function [24]. Nonetheless, Dox is clearly implicated with DITP and VTE and encourages the use of an effective anti-platelet agent to relieve these burdens imposed by it. 

Danshen, the dried root of the *Salvia miltiorrhiza* herb, is a traditional Chinese medicine that has been historically used throughout Asia for centuries to treat cardiovascular disease. Salvianolic acid (SA) compounds, e.g., salvianolic acids A, B, and C (SAA, SAB, and SAC, respectively), represent the major extracts and active components of Danshen and have recently been recognized for inhibitory effects on platelet function [25,26]. For example, SAA inhibits platelet activation and arterial thrombosis, SAB inhibits ADP-induced platelet aggregation and adhesion to collagen by interfering with receptor α2β1, and a combination of SAA and SAC synergistically inhibits ADP- and thrombin-induced platelet aggregation [25,27,28]. However, whether SA compounds are viable therapies to inhibit Dox-associated thrombocytopenia and thrombotic risk by interfering with platelet function has not been determined.

In the present study, we investigated the direct influence of Dox on platelets including activation, clearance, and intracellular signalling pathways, as well as the ability of SAC to attenuate these effects. We demonstrated that Dox directly induced platelet activation and augmented platelet-cancer cell interaction. Notably, SAC suppressed the aforementioned adverse effects of Dox. Our study supports SAC as a promising therapy in conjunction with Dox to reduce the associated risk of DITP, VTE, and the repercussions of inducing pro-cancer platelet pathology in the tumor microenvironment.

## 2. Results

### 2.1. Dox Induced Platelet Activation and Degranulation Inhibited by SAC

**Figure 1:**
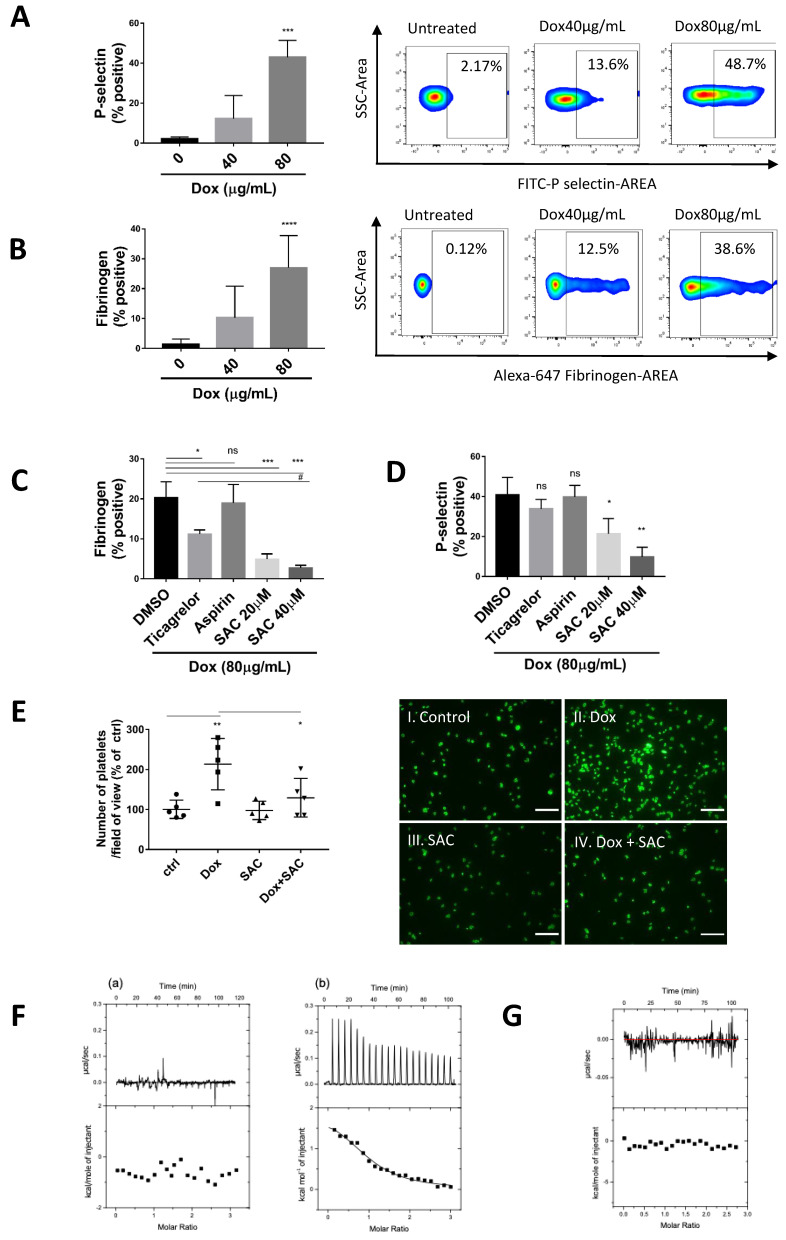
Dox-induced Platelet Activation Inhibited by SAC. Diluted mouse PRP was incubated with Dox for 25 min at 37 °C. Platelet activation was determined by flow cytometry using anti-P-selectin antibody (**A**) and Alexa 647-conjugated Fibrinogen binding (**B**). Data are shown as the percentage of positive platelets. (**C**,**D**) Effects of pretreatment with P2Y12 inhibitor Ticagrelor (0.1 µM), TxA2 inhibitor Aspirin (6 mM), or SAC (20 µM, 40 µM) on Dox (80 µg/mL)-induced murine platelet activation measured by P-selectin (**C**) and Fg binding (**D**) with flow cytometry. (**E**) Murine platelet spreading over immobilized fibrinogen examined by confocal imaging and number of adherent platelets per field of view quantified using Analyze Particles function in ImageJ/Fiji software. Scale bars = 64 μm (40×. Representative images of 3 separate experiments displayed. n ≥ 3 for all experiments. (**F**) ITC thermogram showing dissociation of (**a**) 80 µg/mL doxorubicin and (**b**) 160 µg/mL doxorubicin in Tyrode buffer. (**G**) ITC thermogram showing no interaction between fibrinogen and doxorubicin. For each, the top shows the raw titration data showing the heat resulting from each injection of ligand into buffer solution. The bottom shows the integrated heat plot after correcting for the heat of dilution. Data displayed as mean ± SD. Statistical significance determined by one-way ANOVA. (# *p* < 0.05, * *p* < 0.05, ** *p* < 0.01, *** *p* < 0.001, and **** *p* < 0.0001, ns = not significant). PRP = platelet-rich plasma, Fg = fibrinogen, Ctrl = control.

Several studies indicated a possible involvement of platelets in the increased thrombotic risk in patients following Dox treatment. However, the reported effects of Dox on platelet function remain controversial, and the precise mechanism remains unclear [21,22,23,24,29,30]. To assess whether platelets are activated following Dox treatment, the percentage of CD62P (P-selectin)-positive and Fibrinogen (Fg)-positive platelets were evaluated using flow cytometry. We found that Dox dose-dependently increased P-selectin expression and soluble Fg binding in murine (Figure 1A,B) and human platelets (Figures S1 and S2), demonstrating Dox-induced α granule release and αIIbβ3 activation. 

As predominant anti-platelet drugs, we tested aspirin and ticagrelor against Dox-induced platelet activation. At the concentration to effectively inhibit ADP-induced activation (Figures S4 and S5), aspirin could not inhibit Dox-induced activation, and ticagrelor exhibited a moderate effect as evidenced by platelets binding to Fg (Figure 1C) but no inhibition of P-selectin expression (Figure 1D). Comparatively, SAC significantly reduced platelet activation as indicated by the same markers (Figure 1C,D). The partial inhibition by ticagrelor may be attributed to inhibiting released ADP stored in platelet-dense granules from further stimulating platelets in an autocrine and paracrine fashion. Moreover, we observed platelet adhesion over immobilized fibrinogen, which was revealed by FITC-conjugated phalloidin staining to visually corroborate the effects of Dox and SAC on platelet activation. The number of platelets adhered to immobilized Fg was approximately two-fold higher after incubation with Dox compared to basal adhesion levels and was reduced in the presence of SAC (Figure 1E). 

Previous reports have indicated that salt and other solvent conditions can cause Dox to form aggregates [31,32,33,34], which can be monitored by isothermal titration calorimetry (ITC) and visible absorption spectroscopy methods. To exclude the possibility of Dox aggregates crosslinking platelets to induce activation, we confirmed that Dox was monomeric at the concentration and conditions used in these experiments. The ITC titration curve showed no dissociation heat at 80 µg/mL of Dox, whereas titration of 160 µg/mL of doxorubicin produced an endothermic profile due to the dissociation of aggregates (Figure 1F). These results were further confirmed by visible absorption spectroscopy. Molar absorptivity at 497 nm could be discerned from that at 480 nm, indicating the presence of a monomer at 80 µg/mL Dox. Molar absorptivity at 480 nm and 497 were indistinguishable, indicating dimerization/aggregation at 160 µg/mL Dox (Figure S3).

We also excluded the interaction between Dox and Fg that may contribute to the observed platelet Fg binding (Figure 1G). Taken together, these data clarify that Dox induced platelet activation, which was inhibited by SAC.

### 2.2. SAC Inhibited Cancer-Platelet Interaction Enhanced by Dox

Activated platelets have been shown to interact with cancer cells and promote metastasis [35,36,37,38]. We hypothesized that induction of platelet activation by Dox could enhance platelet-tumor cell interactions. Breast cancer remains one the most prevalent cancer diagnoses worldwide, for which Dox is a first-line therapy [39], particularly for triple-negative breast cancer (TNBC) due to its well-documented effectiveness. However, multiple studies have reported an increased risk of thrombosis for breast cancer patients receiving Dox treatment [40,41]. With these considerations, we focused on two TNBC models to examine cancer-platelet interaction. 

**Figure 2:**
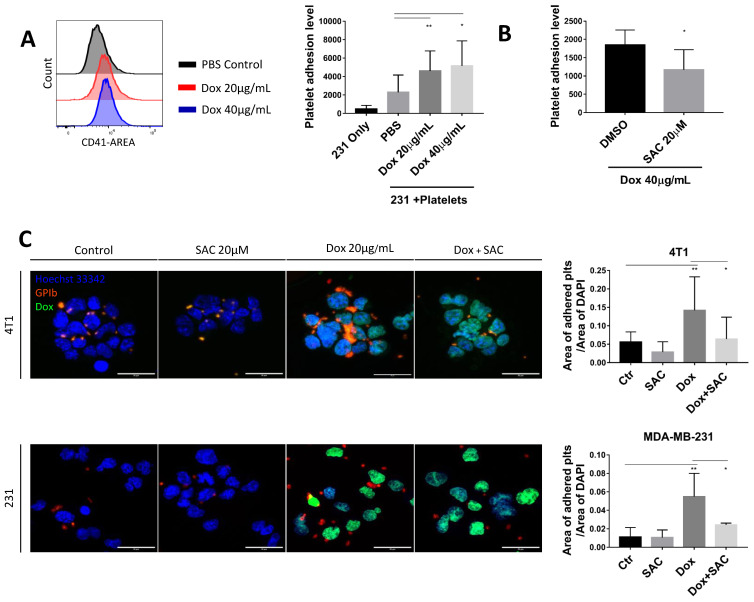
Dox Enhances cancer cell-platelet Interaction. (**A**,**B**) Platelet adhesion levels to MDA-MB-231 cells stained for platelet marker CD41 and quantified by flow cytometry. (**C**) Confocal imaging of breast cancer cell lines (4T1, upper row and MDA-MB-231, lower row) interacting with murine platelets. Cell nuclei were stained with Hoechst 33342 (blue); platelets were stained with GPIbβ (red); and Dox exhibited autofluorescence (green). The level of platelet-cancer cell interaction was shown as GPIbβ fluorescence area per area of Hoechst. Representative images chosen from 3 separate experiments. Scale bars = 30 μm. The fluorescence area was quantified using the Area Quantification FL module on HALO^®^ platform. Data are displayed as mean ± SD. n ≥ 3 for all experiments. Statistical significance was determined by one-way ANOVA (**A**,**C**) or paired *t*-test (B). (* *p* < 0.05, ** *p* < 0.01). Ctr = Control, 231 = MDA-MB-231.

Untreated or Dox-treated platelets were incubated with breast cancer cell line MDA-MB-231, then unbound platelets were washed, and cancer cells were analyzed for platelet-specific marker CD41 to indicate platelet adhesion. Platelet signal on MDA-MB-231 cells was increased in the groups of 20 µg/mL and 40 µg/mL of Dox (4603 ± 2174 and 5151 ± 2718, respectively) versus control (2300 ± 1867) (Figure 2A). Meanwhile, SAC pretreatment reduced this binding by approximately 30% (Figure 2B). Additionally, we used confocal fluorescence imaging to further visualize our observations. Murine platelets were incubated with breast cancer 4T1 (Figure 2C, upper panel) and MDA-MB-231 cells (Figure 2C, lower panel). Then, platelets were stained for GPIbβ (red), cancer cells were stained for Hoechst 33342 (blue), and Dox exhibited autofluorescence (green). By quantifying the area of adhered platelets per area of cell nuclei, we confirmed the enhancement of cancer-platelet interaction by Dox that could be ameliorated by SAC (Figure 2C). Intriguingly, the Dox-treated platelets appeared to be microaggregated as opposed to a sparser distribution in the untreated control groups, suggesting that Dox may aid tumor cell-induced platelet aggregation (TCIPA). Moreover, the groups of SAC alone also appeared to have fewer platelets neighboring the cancer cells, suggesting basal inhibition by SAC beyond Dox-facilitated interaction, possibly through the inhibition of platelet activation induced by ADP released by tumor cells. 

### 2.3. SAC Protected Platelets from Macrophage Phagocytosis 

**Figure 3:**
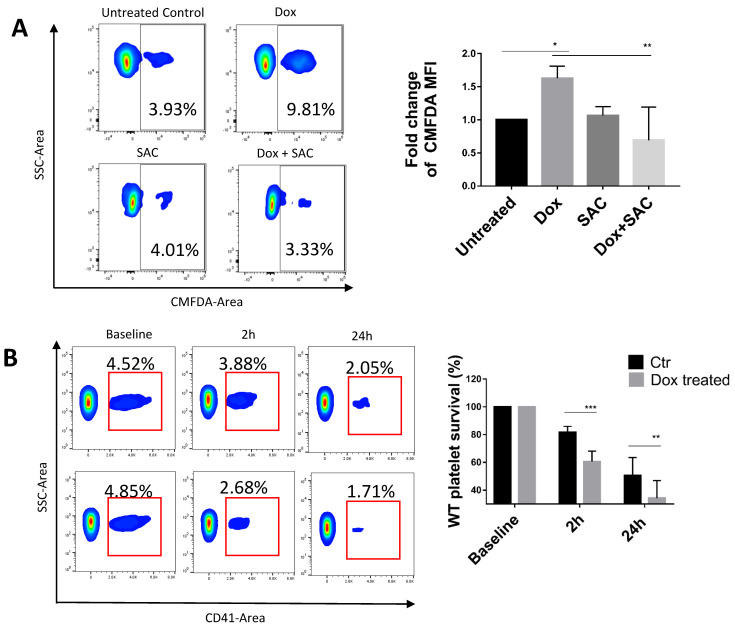
SAC Reduces Platelet Phagocytosis by THP-1 Macrophages. (**A**) Dox increased human platelet phagocytosis by differentiated THP-1 macrophages. Platelets were stained with CMFDA and then co-incubated with differentiated THP-1 cells and 40 μg/mL Dox pretreated with or without SAC (40 µM). THP-1 cells were washed to remove platelets and analyzed for CMFDA signal by flow cytometry. (**B**) Effect of Dox in ex vivo platelet transfusion survival assay. Platelets from WT mice were treated with 40 μg/mL Dox prior to transfusion into CD41^−/−^ mice. Blood samples were taken from recipient mice at 0, 2, and 24 h after transfusion, and platelets were stained for CD41 to determine surviving percentage of transfused platelets. Data displayed as mean ± SD. Statistical significance determined by two-way ANOVA. (* *p* < 0.05, ** *p* < 0.01, *** *p* < 0.001). n ≥ 3 for all experiments. WT = wild type, Ctr = control.

Platelets frequently undergo clearance following normal senescence or activation. One facet of clearance is platelet phagocytosis, which often occurs following macrophage recognition of exposed platelet P-selectin and PS exposure [42]. To evaluate the extent of platelet phagocytosis, platelets were fluorescently labelled and treated with Dox before being introduced to differentiated THP-1 cells. Compared to untreated platelets, when platelets were incubated with Dox, the macrophages exhibited a 1.5-fold increase in the platelet fluorescence signal, and SAC pretreatment resulted in amelioration of clearance. SAC by itself did not inhibit basal phagocytosis, indicating that it does not impair macrophage phagocytic activity (Figure 3A). 

To investigate whether platelets exposed to Dox have accelerated clearance in vivo, we performed platelet survival assays in which 1.5 × 10^8^ WT platelets were transfused into CD41-deficient syngeneic mice, allowing us to distinguish the surviving percentage of WT platelets per time point using an anti-CD41 antibody. At 2 h and 24 h from baseline, the percentage of surviving Dox-treated platelets was 60.59% ± 7.452% and 50.55% ± 12.91%, respectively, compared to 81.59% ± 4.326% and 34.23% ± 12.68% for untreated platelets (Figure 3B), indicating that Dox induced faster clearance of circulating platelets.

### 2.4. SAC Reduced Dox Uptake by Platelets 

**Figure 4:**
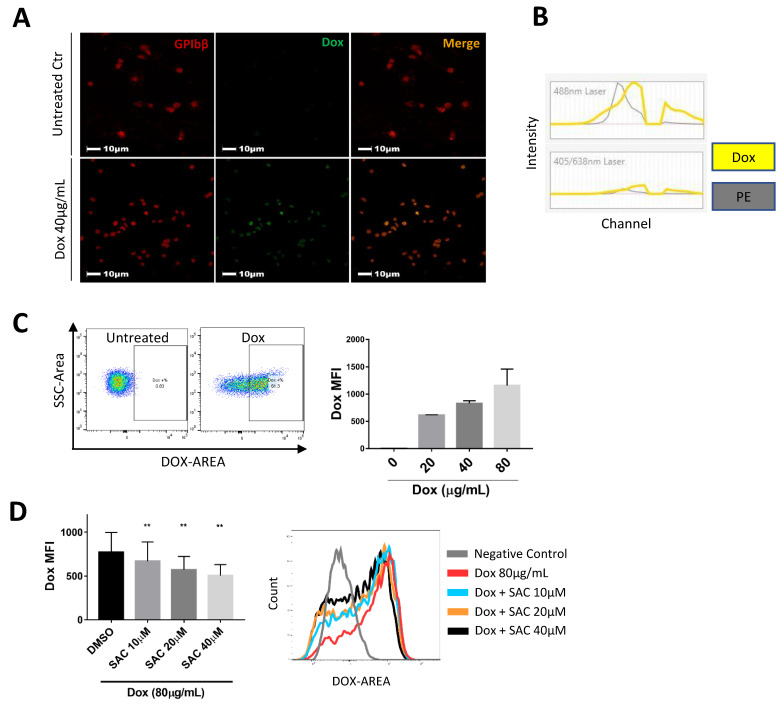
SAC Reduces Dox Uptake by Platelets. (**A**) Confocal imaging visually depicts platelet intake of Dox following incubation. Murine platelets labelled with AlexaFluor-647-conjugated anti-GPIbβ antibody (red, left column) were untreated (upper row) or incubated with Dox (lower row). Dox exhibited autofluorescence ~590nm (Green, middle column). Representative images taken from 3 independent experiments, scale bars = 10 μm. (**B**) Unique Dox spectral signature across all channels determined from spectral cytometer. (**C**) Dose-dependent uptake of Dox by platelets quantified by Dox MFI (right), and representative population shift for platelets exhibiting Dox signal following incubation with 40 μg/mL Dox (left). (**D**) SAC dose-dependently reduced Dox permeation of platelets. Data are displayed as mean ± SD. n ≥ 3 for all experiments. Statistical significance determined by one-way ANOVA. (** *p* < 0.01).

Next, we analyzed Dox permeation of platelets through a combination of fluorescence imaging and flow cytometry. Murine platelets labelled with AlexaFluor-647-conjugated anti-GPIbβ antibody (red) were untreated (Figure 4A, upper row) or incubated with Dox (Figure 4A, lower row). Treated platelets emitted a distinct Dox autofluorescence signal that was absent in untreated platelets, suggesting that Dox accumulates in platelets (Figure 4A, bottom). 

Dox autofluorescence has previously been characterized in conventional cytometers using bandpass filters that capture its peak emission ~590nm. In order to further understand Dox and platelets in our SP6800 spectral cytometer system, we established the spectral signature of Dox across all channel ranges to create a reference spectrum, allowing us to capture its complete signal and improve unmixing from other fluorochromes. The spectra showed a maximum emission in the range of 581–598nm, similar to PE’s spectrum (Figure 4B). Platelets exhibited a Dox-specific signal following incubation, with the MFI increasing in a dose-dependent manner (Figure 4C). Meanwhile, platelets treated with SAC exhibited significantly reduced relative amounts of the Dox signal, with a maximum reduction of 39.52% for 40 μg/mL Dox (Figure S6) and 34.5% for 80 μg/mL Dox compared to respective vehicle controls (Figure 4D).

### 2.5. SAC Ameliorated Dox-Induced Increase in Tyrosine Phosphorylation of Platelet Protein 

**Figure 5:**
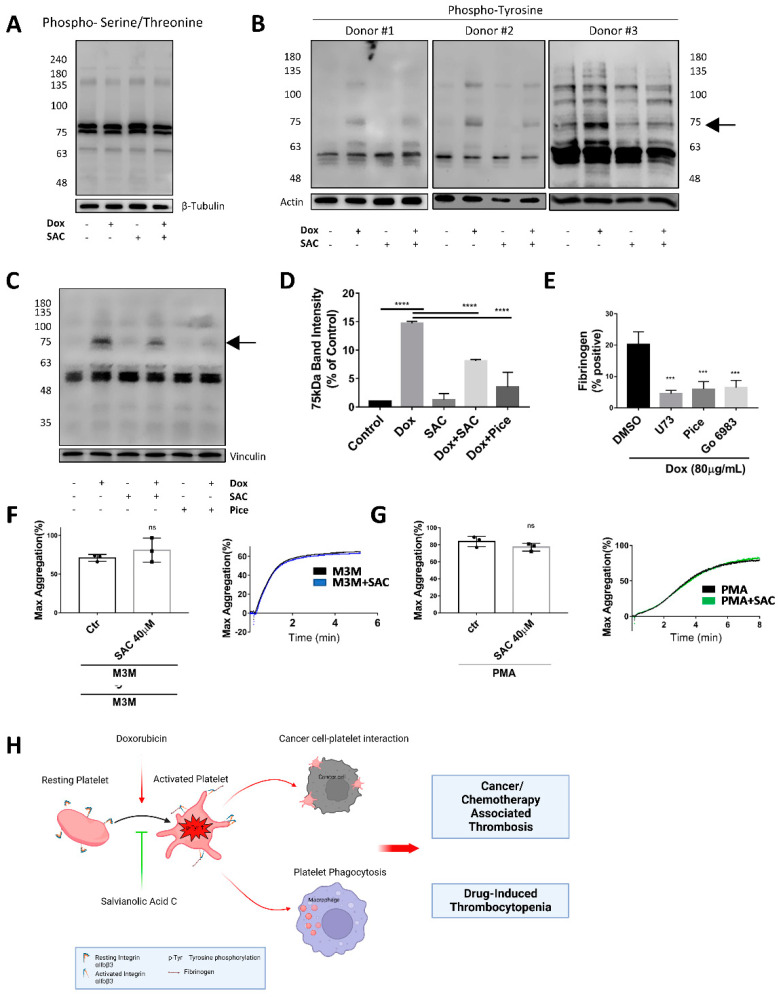
SAC Ameliorated Dox-Induced Tyrosine Phosphorylation of Platelet Proteins. Human gel-filtered platelets were pretreated with SAC (10 µM) or SYK protein inhibitor piceatannol (10 µg/mL) for 2 min before incubation with Dox (40 µg/mL) for 20 min. Total platelet lysate for each group was resolved with SDS-PAGE and probed with anti-phosphoserine/threonine (**A**) or anti-pY (**B**,**C**) antibody. β-tubulin, actin, and vinculin served as loading controls. (**D**) Quantification of band intensity for 75 kDa protein in preceding Western blots. (**E**) Effects of PLC inhibitor U-73122 (2 µM), Syk inhibitor piceatannol (20 μg/mL), or PKC inhibitor Go 6983 (10 μM) on Dox-induced murine platelet Fg binding. (**F**,**G**) SAC (40 µM) on PLC agonist m-3M3FBS (400 µM) and PKC agonist PMA (50 ng/mL) induced human washed platelet aggregation. Aggregation traces are representative of data from at least three independent experiments. (**H**) Schematic summary of novel mechanism for doxorubicin-associated thrombosis and drug-induced thrombocytopenia. Dox induced integrin ⍺IIbβ3 activation and subsequent fibrinogen binding, possibly through upregulated intracellular tyrosine phosphorylation. As a result, Dox could amplify cancer-platelet interaction and facilitate cancer/chemotherapy-induced thrombosis. Dox-treated platelets were also cleared more rapidly by phagocytosis. Salvianolic acid C inhibited Dox-induced platelet activation/cancer-platelet interaction/phagocytosis, which are contributing factors for Dox-associated thrombosis and thrombocytopenia. Created with BioRender.com. n ≥ 3 for all experiments. Data displayed as mean ± SD. Statistical significance determined by one-way ANOVA (**D**,**E**) or paired *t*-test (**F**,**G**). (*** *p* < 0.001, and **** *p* < 0.0001, ns = not significant). U73 = U-73122, Pice = piceatannol, pY = phosphotyrosine, M3M = m-3M3FBS.

Platelet activation signal transduction pathways involve a wide array of protein phosphorylation, of which phosphorylated tyrosine (pY) or phosphorylated serine/threonine (pS/T) represent the two predominant signal transduction mechanisms to convert extracellular stimuli into a functional response [43,44,45]. Probing for general S/T phosphorylation yielded no observable Dox-associated changes in protein phosphorylation (Figure 5A); however, when exploring overall tyrosine residue phosphorylation, we noted a consistent and prominent increase in Dox-treated platelets compared to control, especially of a protein ~75kDa. The density of these bands was reduced in platelets pretreated with SAC, illustrating that SAC could inhibit the Dox-induced phosphorylation (Figure 5B,D). We suspected the ~75kDa band to be spleen tyrosine kinase (SYK), a kinase with a reported molecular weight of 72kDa and heavily associated with the phospholipase C (PLC) and protein kinase C (PKC) axis that can lead to integrin αIIbβ3 inside-out signalling and platelet activation [46]. We treated platelets with SYK-selective tyrosine inhibitor piceatannol and discovered complete abolition of the ~75kDa band in Dox- and piceatannol-treated platelets (Figure 5C,D). This trend was consistent for all individual platelet donor iterations of this experiment, suggesting the involvement of SYK in Dox-induced platelet activation along this axis.

We further investigated the mechanism of Dox-induced platelet activation by including kinase-specific inhibitors or activators along the SYK-PLC-PKC axis and analyzing platelet activation or aggregation. PLC inhibitor U-73122 at 2 µM, SYK inhibitor piceatannol at 20 µg/mL, and PKC inhibitor Go 6983 at 10μM inhibited Dox-induced Fg binding to platelets, implicating the SYK-PLC-PKC axis’ contribution to Dox-induced platelet activation (Figure 5E). However, SAC was unable to inhibit platelet aggregation induced by PLC agonist M3M (Figure 5F) and PKC agonist PMA (Figure 5G), suggesting that SAC acts upstream of PLC and PKC. 

## 3. Discussion

Previous investigations examining Dox and platelet activity have reported inconsistent findings, with one study showing indirect induction of platelet activation by endothelial cell or vascular injury [21], while other studies using isolated platelets have claimed that Dox has no direct effect on platelet activity [24]. To the best of our knowledge, our discoveries are the first to demonstrate direct Dox-induced platelet αIIbβ3 activation, supported by Fg binding results. Furthermore, we also demonstrated Dox-induced platelet alpha granule release by P-selectin expression. However, an eminent factor of the discrepancy in results may reside with incubation time. An earlier study [24] observed no change in P-selectin and PAC-1 after 3 hours of incubation with Dox, while we observed an increase as early as 20 min after incubation. As a result of the extended treatment window, αIIbβ3 may have returned to a resting conformation, as in the case of platelet de-aggregation that has been widely recognized [47,48,49], and P-selectin may have re-internalized [50], disallowing PAC-1 or anti-P-selectin recognition. 

Dox-induced platelet activation in the tumor microenvironment may lead to an even more pernicious cancer progression, as platelets have a pivotal role in cancer pathology underscored by the consequences of cancer-platelet bidirectional interaction. Platelets are responsible for contributing to tumor angiogenesis and sustaining proliferation and anti-apoptotic signals and are linked to facilitating cancer stem-cell phenotypes [51]. In addition, they elevate cancer metastasis by providing epithelial to mesenchymal transition (EMT) signals while physically shielding the metastatic cells in circulation and simultaneously dampening the immune recognition response [38]. As such, Dox may undermine its own efficacy/effectiveness by augmenting cancer-platelet interaction, which also increases the risk of CAT/VTE and thus has long-term implications for patients during and beyond a Dox regimen. 

Clinically observed side effects, as well as previous findings of Dox-enhanced metastasis and stemness [52,53,54], are consistent with the involvement of platelets in cancer pathobiology. Our findings of augmented platelet activation and interaction with cancer cells by Dox may be the missing link to unite these observations, further highlighting the sagacity of including an anti-platelet agent in conjunction with Dox treatment.

We also demonstrated that platelets exhibit a Dox signal following incubation with it. Dox has previously been characterized for its autofluorescence, with a similar signal to PE and Cy3, at an excitation and emission of ~470/595nm, respectively. Previous studies investigating the effect of Dox on platelet phenotype have used fluorescent probes that share similar spectra to Dox for flow cytometry and confocal imaging without taking autofluorescence into consideration. This may result in an overlap of fluorescent signals and inaccurate readings. For example, studies using PE-GPIbβ to monitor microparticle formation, or one using fluo-3 to measure calcium increase, could have reported a false positive spillover signal from Dox [23]. These studies need to be re-examined with other fluorophores or properly compensate the signal to account for Dox, and future studies should also take this phenomenon into account. Interestingly, we further found that the Dox signal in PBS-EDTA was lower than in PBS (data not shown). EDTA induced dissociation of the membrane αIIbβ3 complexes [55], suggesting that integrin αIIbβ3 could be involved in this process. 

Our ex vivo platelet survival assay and in vitro phagocytosis assays suggest a faster clearance of platelets directly induced by Dox and not only indirectly by endothelial cell injury as previously suggested [21]. A retrospective platelet trajectory study following induction of chemotherapy, nonspecific to Dox, found a transient drop in platelet count that resurged shortly after [56]. Despite distinct clinical evidence associating Dox with DITP, few, if any, animal models have been sufficiently able to recapitulate this phenomenon in vivo. These findings indicate that the observation window is crucial to precisely monitor the effect of Dox on platelets in animal models; platelet count may have already resurged in studies collecting samples one week following Dox treatment, exceeding the window of observable effect. Therefore, a harder, more precise look with a more robust time schedule is necessary to understand exactly when Dox can impinge its effect upon platelets. Specifically, in vivo time-course studies would be prudent, with careful regard to the short-term and long-term effects on platelet activation or thrombus formation and investigation of platelet microaggregates or evidence of transient DITP. Additionally, our THP-1 phagocytosis results could not experimentally distinguish between ingested platelets and surface-adhered platelets. Fluorescence microscopy can be performed to validate platelet phagocytosis by macrophages. 

The major receptors that trigger tyrosine kinase pathways in platelets are GPVI, GPIb-IX-V complex, and CLEC2. They share a number of common downstream signaling molecules, especially in the activation of SYK, PLC, and PKC [57], which were shown to be involved in Dox-induced platelet activation in our study. Thus, which, if any, of these tyrosine kinase-associated receptors Dox directly binds to needs to be further explored. 

Due to the prominent impacts of Dox on platelets, other standard-of-care anti-platelet agents have been proposed, such as aspirin and Clopidigrel. However, these agents are subject to limitation, as both introduce a greater risk of bleeding and drug resistance over time. Clopidigrel has even been shown to reduce the anti-cancer effects of Dox [58,59,60,61]. On the other hand, SA boasts a lower bleeding risk and is currently undergoing clinical trials [28,62]. 

As mentioned, the most abundant compounds in the SA family, SAA, SAB, and SAC, have demonstrated anti-platelet and anti-thrombotic capabilities, albeit at varying dosages. SAA at 100 μM [27] and SAB at 140μM [63] significantly inhibited ADP-induced platelet activation. However, we found that SAC achieved comparable inhibition at a much lower concentration of 40 μM. Furthermore, we also found that SAC inhibited Dox-induced platelet activation more efficiently than SAB (data not shown).

Importantly, previous pharmacokinetic studies of SAs indicate that the working concentration of SAC is achievable in vivo. For example, SAA in Rhesus monkeys following a 10 mg/kg IV bolus demonstrated a plasma concentration of approximately 113.293 mg/L (roughly 288.56 µM) [64]. Another study of SAB in rats demonstrated a maximal plasma concentration of 910 μg/mL (1.8404 mM) following 100 mg/kg IV injection [65]. 

Moreover, a study on SAA toxicity in beagles reported a no-observed-adverse-effect dose of 20 mg/kg via IV injection [66]. Taken together, these findings suggest SAC as a viable candidate for further drug development.

In the present study, we found that SAC alleviated Dox-induced platelet activation and cancer-platelet interaction and enhanced platelet clearance, suggesting SAC as a promising agent to be used in conjunction with Dox chemotherapy. Beyond its anti-platelet function, salvianolic acids have also been shown to provide several protective effects, including cardio-, nephro-, vascular-, and neuro-protective attributes specific to Dox-associated toxicity. Furthermore, it has been reported to ameliorate Dox-associated fibrosis [25,67,68,69]. SAC has been reported to localize and enhance the anti-cancer capacity of Dox [70]; thus, SAC is effective not only for protecting against Dox toxicity and as a thromboprophylaxis tool to reduce the Dox-associated risk of thrombosis but also for improving overall treatment efficacy (Figure 5H). 

In summary, our study improves the current understanding of Dox-induced platelet activation and offers perspective to explain the previous inconsistencies. We suggest a mechanism linking the clinically observed VTE and DITP side effects imposed by Dox. Simultaneously, our results exhibit potential for SAC as a novel anti-platelet therapy to protect patients from chemotherapy-associated thrombosis and thrombocytopenia. Taken together, this study presents insight into developing an anti-platelet combination therapy to relieve the platelet-involved side effects imposed by Dox. 

## 4. Materials and Methods

### 4.1. Materials

Doxorubicin hydrochloride was from ThermoFisher Scientific™ (Cat. No: J64000.MA, Waltham, MA, USA). SAC was from MedChemexpress Co., Ltd. (Cat. No. HY-N0319, Monmouth Junction, NJ, USA). Piceatannol was from Millipore (Burlington, MA, USA). Go 6983 and m-3M3FBS were purchased from Selleck Chemicals, LLC (Cat. No. S0982, Houston, TX, USA). Phorbol 12-myristate 13-acetate (PMA) was from Millipore. Anti-phosphotyrosine antibody, clone 4G10, was obtained from Millipore (Cat. No. 05-321X). Phospho-(Ser/Thr) Phe Antibody was obtained from Cell Signaling Technology (Danvers, MA, USA). All antibodies for flow cytometry were obtained from BioLegend (San Diego, CA, USA). Alexa Fluor™ 647-conjugated fibrinogen from human plasma was purchased from Sigma-Aldrich (Cat. No. F35200, St. Louis, MO, USA). Fibrinogen from human plasma was purchased from Sigma-Aldrich (Cat. No. F3879). Anti-GPIbβ antibodies conjugated with DyLight 649 were from Emfret Analytics (Eibelstadt, Bayern, Germany). CellTracker™ Green CMFDA Dye was from ThermoFisher Scientific™. 

### 4.2. Mice

The line of αIIb KO (αIIb-/-) mice was obtained from the laboratory of Dr. Jon Frampton (Birmingham Medical School, Birmingham, UK) [71] and was backcrossed onto C57BL/6 WT background mice ten times. C57BL/6 wild type (WT) and BALB/c WT mice were purchased from Charles River Laboratories (Montreal, QC, Canada) at 6 to 10 weeks of age. The genotype of the mice was confirmed using polymerase chain reaction analysis. All mice were housed at the Research Vivarium of the Li Ka Shing Knowledge Institute (St. Michael’s Hospital, Toronto, ON, Canada) and cared for by the staff of the facility. All experimental protocols were approved by the Animal Care Committee. 

### 4.3. Platelet Aggregation

Aggregation experiments were performed as previously described [47]. Briefly, human gel-filtered platelets (hGFPs) were incubated with the indicated reagents. Aggregation was measured in an aggregometer (Model 700 Chrono-Log Corporation, Havertown, PA, USA).

### 4.4. Preparation of Human and Mouse Platelets

Blood samples were obtained from healthy donors and collected into vacutainers containing 3.8% (*w*/*v*) sodium citrate. Platelet-rich plasma (PRP) was prepared by centrifuging the whole blood at 300× *g* for 7 min at maximum acceleration and minimum brake. Washed platelets were prepared by adding PBS-EDTA to PRP, centrifuging at 800× *g* for 10 min, and re-suspending in 1× Tyrode buffer (TB) (129 mM NaCl, 0.34 mM Na_2_HPO_4_, 2.9 mM KCl, 12 mM NaHCO_3_, 20 mM HEPES, 5 mM glucose, and 1 mM MgCl_2_ to a final pH of 7.4) to an approximate concentration of 3 × 10^8^ platelets/mL. Mouse blood samples were collected by retro-orbital bleeding, and mouse washed platelets were prepared in the same manner described. 

Human gel-filtered platelets (hGFPs) were prepared by isolating PRP from sodium-citrated whole blood as previously described [49], which was then layered over a column of Sepharose 2B (Sigma-Aldrich) and covered with TB. The column was allowed to drain, and fractions of platelet-containing TB were collected and combined to a final concentration of 2.5 × 10^8^ platelets/mL. 

### 4.5. Dox/SAC Treatment of Platelets 

Prepared platelets were aliquoted to equal amounts per treatment group. SAC and Dox combination groups were first exposed to SAC at the appropriate working concentration for 2–5 min prior to incubation with respective Dox concentration(s) for the predetermined experiment conditions, out of direct light to preserve Dox integrity.

### 4.6. Measurement of Platelet Adhesion to Cancer Cell by Flow Cytometry 

Platelets were pretreated with SAC and Dox before being added to cancer cells (MDA-MB-231, 4T1 cell lines provided by Dr. George M. Yousef), at a ratio of 100:1 for 20 min of incubation. Then, cells were washed by centrifugation (150× *g*, 5 min, 9/3 acceleration/deceleration) and stained for CD41 for analysis by flow cytometry, wherein singlet cancer cells were measured for median fluorescence intensity (MFI) of CD41, correlating to the interaction of platelets bound to cancer cells.

### 4.7. Measurement of Platelet and Cancer Cell Interaction by Immunofluorescent Staining 

Platelets were pretreated with SAC and Dox before being added to cancer cells at a ratio of 100:1 for 15 min of incubation, followed by centrifugation (150× *g*, 5 min, 9/3 acceleration/deceleration) to wash off unbound platelets. Cells were then fixed and stained with Hoechst 33342 and DyLight 649 anti-GPIbβ antibodies and imaged by fluorescence microscopy.

### 4.8. Determination of Platelet Activation by Flow Cytometry

Measurements of P-selectin expression and fibrinogen binding were performed as we previously described [72]. We used diluted PRP treated with drugs as described in each figure legend in the presence of APC anti-human CD62P (P-Selectin) antibody, APC anti-mouse/rat CD62P (P-selectin), and Alexa Fluor™ 647-conjugated fibrinogen individually. Platelets were diluted with PBS-EDTA or washed by centrifugation after 20–30 min of incubation in the dark before analyzing on SP6800 Spectral Analyzer (Sony Biotechnology, Inc, San Jose, CA, USA). Spectral unmixing was ensured to separate individual fluorochrome indices for optimal signal collection.

### 4.9. Platelet Spreading on Immobilized Fibrinogen

Platelet adhesion to immobilized fibrinogen was achieved as we described previously [73,74,75]. Platelets were stained with fluorescence isothiocyanate (FITC)-labelled phalloidin (Cat. No. 40735ES75; Yeasen Biotech Co., Ltd., Shanghai, China) and visualized by Olympus Upright BX50 Microscope. The number of adhered platelets and the platelet surface area were analyzed using ImageJ software v1.53t (NIH, Bethesda, MD, USA).

### 4.10. Preparation of Total Platelet Lysate Protein for Western Blot

Washed human platelets were split into equal amounts, and appropriate groups were pretreated with either SAC or SYK inhibitor piceatannol (10 µg/mL final) for 2 min preceding incubation with Dox for ~25 min. Platelets were lysed by adding 1× and 1 mM of the loading buffer and DTT, respectively, and heated at 75 °C for 20 min. Platelet lysate total protein was loaded and resolved by SDS-PAGE and Western blot. The membrane was probed for total phosphorylated tyrosine protein residues using anti-pY mAb. The membrane was re-probed for intracellular cytoskeleton protein Vinculin as the internal loading control. 

### 4.11. Evaluation of Platelet Phagocytosis

The platelet phagocytosis assay was modified from our previous studies [76,77,78]. Briefly, THP-1 human leukemia cells, purchased from ATCC (TIB-202), were stimulated with ~200 nM PMA to induce differentiation. A total of 24 hours later, deep red-labelled hGFP was prepared and treated with SAC and then aliquoted to rinsed THP-1 cells and covered with RPMI 1640. Dox was added to appropriate wells to the working concentration, and the cells and platelets were allowed to incubate for 60 min at 37 °C. The assay was stopped by placing the plate on ice and washed with cold PBS. Cells were fixed with 4% PFA for 20 min at room temperature and then washed 3 times with cold PBS. Cells were gently scraped for flow cytometry analysis.

### 4.12. Platelet Survival Assay

Isolated platelets from WT donor mice were treated with or without Dox (40 µg/mL) for 30 min at 37 °C and then injected into recipient CD41-deficient mice via tail vein. After transfusion, 10 µL of blood from saphenous bleeding was collected into 240 µL PBS-EDTA at various time points, followed by centrifuging at 150× *g* for 2 min. Then, 50 µL of the supernatant was stained with BV605-CD41, and the percentage of CD41-positive platelets (transfused platelets) was determined by flow cytometry. Data were normalized by designating the percentage of CD41-positive platelets at the first time point (immediately after tail vein injection) as 100% and expressing all other different post-transfusion times as the percentage of this value.

### 4.13. Isothermal Titration Calorimetry

ITC experiments were performed using a MicroCal VP-ITC instrument (Malvern Panalytical, Malvern, UK). Samples were degassed before analysis with a MicroCal Thermo Vac (Malvern Panalytical) unit for 5 min. Titrations were performed with either fibrinogen or Tyrode buffer solution in the cell and doxorubicin, as the titrant, in syringe. All experiments consisted of an initial delay of 60 s, the first injection of 2 µL, and a 300 s delay. Subsequent 20 injections were 14 µL, spaced every 300 s. The first point was removed from all data sets due to the different injection volume and delay parameters. The binding experiments were performed at 25 °C using a fibrinogen concentration of 1.5 µg/mL and a doxorubicin concentration of 40 µg/mL. ITC data were fit to a one-site binding model using the manufacturer-provided Origin 7 software (Malvern Panalytical). Dissociation experiments were performed at 25 °C using doxorubicin concentrations of 80 µg/mL and 160 µg/mL. ITC data were fit to a dissociation model using the manufacturer-provided Origin 7 software.

### 4.14. UV-Vis Measurements

All measurements were obtained on a Carry 100 Bio UV-Vis Spectrophotometer (Siemens Healthineers, Erlangan, Germany) in Tyrode buffer at 25 °C using cells of 1 cm path length. Doxorubicin (80 µg/mL and 160 µg/mL) was prepared in the Tyrode buffer, and the disappearance of maxima at 497 nm was observed.

### 4.15. Statistical Analysis 

FlowJo^TM^ v10.8 Software (BD Life Sciences, Franklin Lakes, NJ, USA) and Sony SP6800 were used to analyze the flow cytometry data. GraphPad Prism (version 8, San Diego, CA, USA) was used for data visualization and analysis. Statistical analysis was performed using one-way ANOVA, two-way ANOVA, or Student’s *t*-test. Results are presented as mean ± standard deviation (SD) with significance defined as * *p* < 0.05, ** *p* < 0.01, *** *p* < 0.001, and **** *p* < 0.0001, ns = not significant. 

## Data Availability

Data are contained within the article and Supplementary Materials.

## Supplementary Materials

The following supporting information can be downloaded at: https://www.mdpi.com/article/10.3390/ph15121444/s1, Figure S1: Dox-Induced Human Platelet Fibrinogen Binding Inhibited by SAC; Figure S2: Dox-Induced Human Platelet P-selectin Expression Inhibited by SAC; Figure S3: Doxorubicin Visible Absorption Spectra; Figure S4: Ticagrelor and Aspirin Inhibit ADP-Induced Platelet Activation; Figure S5: SAC Inhibits ADP-Induced Platelet Activation; Figure S6: SAC Reduces Dox Permeation of Platelets.
