# Supplementary material for: Doxorubicin-Induced Platelet Activation and Clearance Relieved by Salvianolic Acid Compound: Novel Mechanism and Potential Therapy for Chemotherapy-Associated Thrombosis and Thrombocytopenia
Source: Pharmaceuticals (Basel). 2022 Nov 22;15(12):1444. doi: 10.3390/ph15121444 (PMC9788583; doi:10.3390/ph15121444)
Supplement: Supplementary file 1 [file pharmaceuticals-15-01444-s001.zip › pharmaceuticals-1986090-supplementary.pdf]

Figure S1.

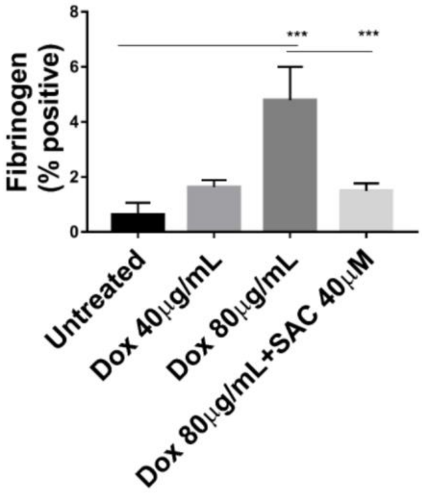

Figure S1. Dox-Induced Human Platelet Fibrinogen Binding Inhibited by SAC. Diluted human PRP was incubated with Dox for 25 min at 37oC. Platelet activation was determined by flow cytometry using Alexa 647-conjugated Fibrinogen binding. Data are displayed as mean ± SD. (*** $p \leq 0.001$) n ≥ 3.

Figure S2.

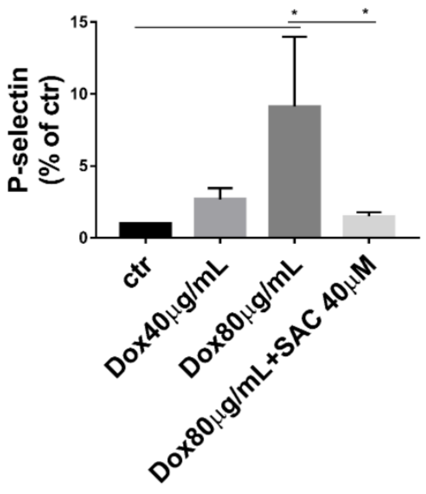

Figure S2. Dox-Induced Human Platelet P-selectin Expression Inhibited by SAC. Diluted human PRP was incubated with Dox for 25 min at 37oC. Platelet activation was determined by flow cytometry using anti-P-selectin antibody. Data are displayed as mean ± SD. (* $p \leq 0.05$) $n \geq 3$. Ctr = Control.

Figure S3.

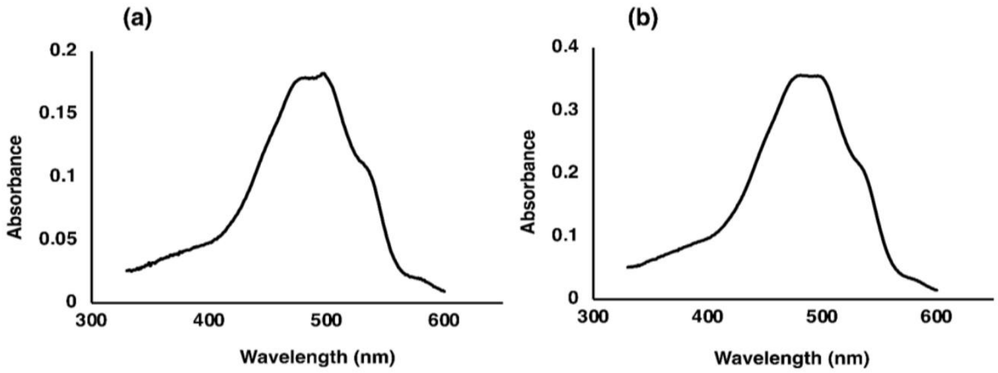

Figure S3. Doxorubicin Visible Absorption Spectra. Representative images depicting the visible absorption spectra of Dox at 80 μg/mL (a) and 160 μg/mL (b) in Tyrode buffer at 25 °C.

Figure S4.

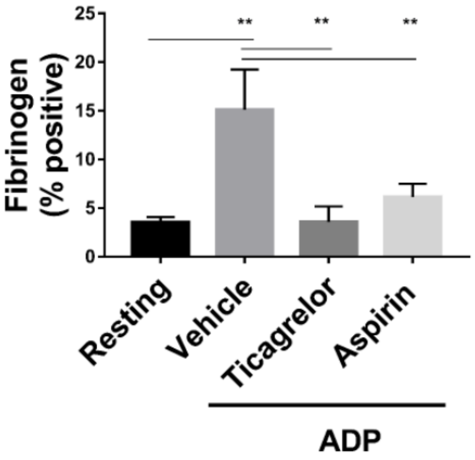

Figure S4. Ticagrelor and Aspirin Inhibit ADP-Induced Platelet Activation. Effects of pretreatment with P2y12 inhibitor Ticagrelor (0.1 μM) and TxA2 inhibitor Aspirin (6 mM) on ADP (1 μM)-induced murine platelet activation, measured by Alexa 647-conjugated Fibrinogen binding with flow cytometry. Data are displayed as mean ± SD. (** $p \leq 0.01$) n ≥ 3.

Figure S5.

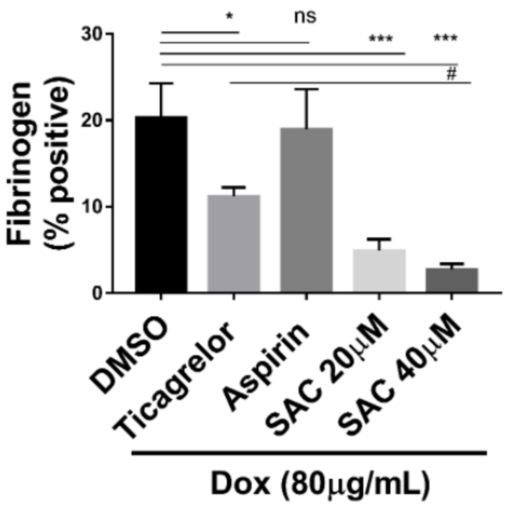

Figure S5. SAC Inhibits ADP-Induced Platelet Activation. Effects of SAC pretreatment on ADP (1 μM)- induced murine platelet activation, determined by flow cytometry using Alexa 647-conjugated Fibrinogen binding. Data are displayed as mean ± SD. (# $p \leq 0.05$ * $p \leq 0.05$, *** $p \leq 0.001$, ns = not significant) n ≥ 3.

Figure S6.

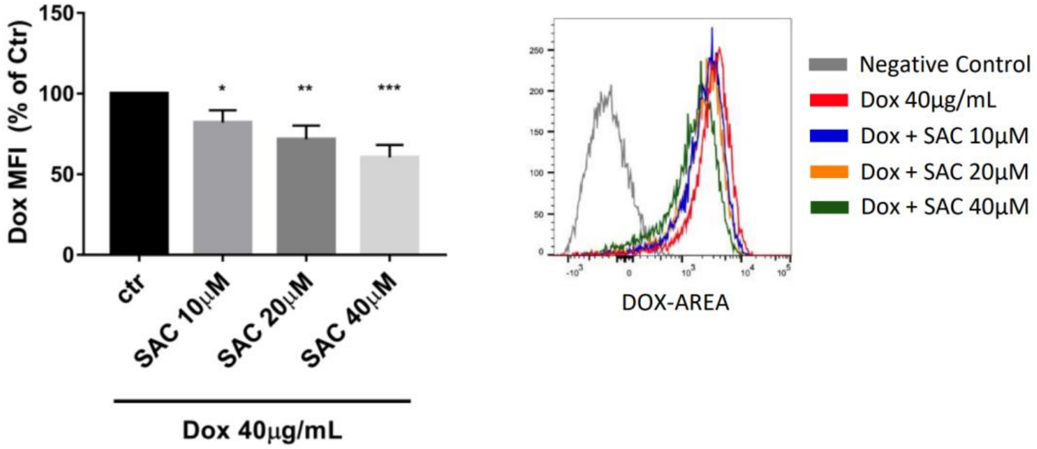

Figure S6. SAC Reduces Dox Permeation of Platelets. Platelets pretreated with SAC exhibited reduced permeation of Dox incubated at 40 μM. Data are displayed as mean ± SD. (* $p \leq 0.05$, ** $p \leq 0.01$, *** $p \leq 0.001$) n ≥ 3.
